# SAMSN1 restricts Japanese encephalitis virus replication by recruiting TRIM21 to degrade viral proteins and activate the interferon pathway

**DOI:** 10.1186/s13567-026-01822-x

**Published:** 2026-09-22

**Authors:** Chen Wang, Wenzhen Qin, Xingya Wang, Xinyu Yang, Wu Tong, Hai Yu, Guangzhi Tong, Tongling Shan, Ning Kong, GuangXu Xing, Hao Zheng

**Affiliations:** 1 Shanghai Veterinary Research Institute, Chinese Academy of Agricultural Sciences, Shanghai, China; 2 Jiangsu Co-Innovation Center for the Prevention and Control of Important Animal Infectious Disease and Zoonoses, Yangzhou University, Yangzhou, China; 3 Institute for Animal Health, Key Laboratory of Animal Immunology of the Ministry of Agriculture, Henan Academy of Agricultural Sciences, Zhengzhou, China

**Keywords:** Japanese encephalitis virus, SAMSN1, IFN, TRIM21

## Abstract

Japanese encephalitis virus (JEV), a zoonotic mosquito-transmitted flavivirus, induces human encephalitis and swine reproductive diseases. No antiviral drugs or treatments exist for flavivirus infections, which cause severe symptoms. Even though some host proteins can suppress flavivirus proliferation, the virus can inhibit this antiviral effect. This study identified a host protein SAMSN1 (SAM domain, SH3 domain, and nuclear localization signal 1; also referred to as HACS1, SLY2, and NASH1) having antiviral activity during JEV replication. SAMSN1 was upregulated during JEV infection. The expression of SAMSN1 inhibits JEV replication by binding and degrading JEV-encoded nonstructural proteins (NS1, NS5) through the proteasome and the SAMSN1-TRIM21-p62 autophagy protein degradation pathways. SAMSN1 induced expression of type I interferon through the RIG-I/MyD88-TBK1-IRF3 axis in the JEV infection process. Current findings reveal a novel role of SAMSN1 in inhibiting JEV replication and proliferation, through viral protein degradation and interferon pathway upregulation.

**Supplementary Information:**

The online version contains supplementary material available at https://doi.org/10.1186/s13567-026-01822-x.

## Introduction

Japanese encephalitis (JE), a mosquito-borne pathogen encephalitis, occurs primarily in southern and eastern Asia [1]. Japanese encephalitis virus (JEV) was first discovered in Japan in 1870 [2]. The virus is spread among pigs or birds by mosquitoes [3], and the final hosts include humans and other mammals such as horses [4]. The lack of effective anti-JEV therapies and high mortality rates in humans make it a global health concern [5].

JEV is a flavivirus from the *Flaviviridae* family. It has a single-strand positive-sense RNA genome of approximately 10–11 kilobases (kb). At the 5’ end, the JEV genome encompasses a type I cap. Its 3’ end is devoid of the poly-A tail; instead, it has a large open reading frame (ORF) flanked by 5’ and 3’ untranslated regions (UTRs). Following cytoplasmic discharge, the genome is directly translated into a single polyprotein precursor, cleaved into seven nonstructural proteins (NS1, NS2A, NS2B, NS3, NS4A, NS4B, and NS5) and three structural proteins (C, pre-membrane [prM], and envelope [E]) [6]. The flavivirus NS1 is a highly conserved glycoprotein existing in diverse forms. Cell surface-related and secreted NS1 are highly immunogenic, which trigger host pathogenesis of flavivirus infection [7]. NS5 is a multifunctional protein. It has a C-terminal RNA-reliant RNA polymerase (RdRp) module as well as an N-terminal methyltransferase (MTase) domain. The RdRp domain has a conserved GDD motif exerting a predominant effect on RNA amplification [8, 9]. Structural proteins are essential for viral entry and virion formation, while NS proteins are required for host cell invasion and viral replication [10]. As members of the genus *Flavivirus*, the nonstructural proteins NS1 and NS5 exhibit complex interaction networks with the autophagic pathway. NS1 regulates autophagy through multiple mechanisms and participates in viral pathogenesis. Notably, secreted NS1 and NS1′ from JEV have been shown to induce degradation of tight junction proteins via the autophagic–lysosomal pathway, resulting in increased permeability of human cerebral microvascular endothelial cells [11]. In contrast, NS5 remains relatively understudied in the context of autophagy. Regarding the regulation of mitophagy, NS5 interacts with the mitochondrial trifunctional protein (MTP), thereby disrupting long-chain fatty acid metabolism, inducing mitochondrial dysfunction, and indirectly activating mitophagy [12].

Macroautophagy or autophagy is a primary pathway for degrading cellular constituents via lysosome hydrolases. It is activated due to cellular stress to maintain cellular homeostasis. The ubiquitin–proteasome system (UPS) is another main intracellular degradation pathway. Here, the proteasome identifies and degrades proteins labeled by polyubiquitin chains [13]. Autophagy, a highly conserved catabolic event, enables cellular self-digestion [14]. The virus–autophagy interactions are extremely intricate [15]. As a host defense strategy, autophagy can be induced to antagonize viral infections. The viral components or cytoplasmic virions are delivered to lysosomes to be degraded. This stimulates the inflammatory reaction, antigen presentation, pathogen recognition, and elimination [16]. Galectin-9 suppresses hepatitis B virus replication through the selective autophagy of viral proteins mediated by p62/SQSTM1 [17]. Previous studies have demonstrated that certain viruses can restrict or escape autophagy, while some can even hijack the autophagic machinery or circumvent the host immunity. For instance, in the foot-and-mouth disease virus, VP1 achieves autophagy-mediated YTHDF2 degradation to regulate interferon regulatory factor 3 (IRF3) activity for its replication [18].

SAMSN1 (SAM domain, SH3 domain, and nuclear localization signals 1; also called HACS1, SLY2, and NASH1) is a signaling adaptor protein expressed in hematopoietic tissues. It is found to have lower expression levels in cardiac, cerebral, placental, and pulmonary tissues [19]. This protein is often used in the translocation process of hematopoietic malignancies and is associated with frequent heterozygous deletions in pulmonary carcinoma cells [20]. SAMSN1 also exerts a vital impact on the activation and differentiation of β cells [21], and it is engaged in pulmonary carcinoma as a tumor inhibitor gene [22], implying its close correlation with immune disorders and cancer. There are very few reports of SAMSN1 virulence. During our initial screening for host factors interacting with the JEV nonstructural proteins, we identified SAMSN1 as a novel interacting partner. Subsequent functional studies revealed that SAMSN1 significantly modulates JEV replication. Consequently, we focused on elucidating the underlying molecular mechanism by which SAMSN1 influences viral replication. SAMSN1 degraded JEV NS1 and NS5 proteins through autophagy and proteasome protein degradation pathways, hindering viral replication. SAMSN1 activates the interferon (IFN) signaling pathway via upregulation of myeloid differentiation factor 88 (MyD88) and retinoic acid-inducible gene protein I (RIG-I). The SAMSN1-TRIM21-NS1(NS5)-p62 is a novel pathway for SAMSN1 in antiviral reaction regulation.

## Materials and methods

### Cells and viruses

Dulbecco’s modified Eagle medium supplemented with 10% fetal bovine serum (FBS) was used to culture HEK 293 T cells (ATCC, CRL-11268). The cell lines were subjected to incubation at 37 °C in a 5% CO_2_ atmosphere for both cell lines. JEV HEN0701 strain (GenBank: FJ495189.1) was stored at the CAAS Shanghai Veterinary Institute. Experiments involving viral infections were conducted in a biosafety level 2 (BSL-2) laboratory setting.

### Plasmids and transfection

Plasmid construction was performed using Flag-SAMSN1 as an example: First, we obtained the target gene sequence from NCBI and optimized it. Using Primer 5 software, primers were designed based on vector maps including p3×Flag-CMV-7.1, pCAGGS, and pCold-TF. The tag position is typically added at the N-terminus. Homologous recombination cloning was performed using the Vazyme ClonExpress II One Step Cloning Kit. The resulting plasmid was subsequently extracted using the Omega Plasmid Mini Prep Kit and stored at −20 °C. When confluence reached 80–90%, the recombinant plasmid was transfected into cells using Lipofectamine 3000 reagent (Invitrogen: L3000015). Small interfering RNAs (siRNAs) were transfected into HEK 293 T/PK15 cells using Lipofectamine RNAiMAX reagent (Invitrogen:13778150).

### Antibodies and reagents

Bafilomycin A1 (Baf A1; 54645), MG132 (M7449), chloroquine phosphate (CQ; PHR1258), and 3-methyladenine (3-MA; M9281) were procured from Sigma-Aldrich. JEV NS3 rabbit antibody (44958) was obtained from GeneTex. Anti-MYC-tag antibody (2276S) and anti-FLAG M2 antibody (F1804) were acquired from Sigma-Aldrich. HA-tag rabbit antibody (51 064–2-AP), anti-GAPDH mouse antibody (60004-1-Ig), anti-RIG-I mouse antibody (67556-1-Ig), anti-MyD88 rabbit antibody (23230-1-AP), anti-glutathione S-transferase (GST)-tag mouse antibody (HRP-66001), horseradish peroxidase (HRP)-conjugated anti-mouse (SA00001-1), and anti-rabbit (SA00001-2) IgG antibodies were procured from Proteintech. The siRNA was prepared by GenePharma (Table 1).

**Table 1:** **List of primer and siRNA sequences used in this study**

Purpose	Names	Sequence (5’-3’)
Real-time polymerase chain reaction (PCR)	*pSAMSN1* forward	AGTTCAGATGTGGAACAGTTGG
	*pSAMSN1* reserve	TCCGAAAACGATCAAAATTCCCA
	*pGAPDH* forward	ATGGATGACGATATTGCTGCGCTC
	*pGAPDH* reverse	TTCTCACGGTTGGCTTTGG
Primers	JEV *NS3* forward	GGACACCACCACAGGAGTTT
	JEV *NS3* reverse	ATAGCTTATGCGGTCTTCCTTCAC
siRNA sequences	*si-SAMSN1* sense	GAUUCACCUUCAGGAAUAUTT
	*si-SAMSN1* antisense	AUAUUCCUGAAGGUGAAUCTT
	NC sense	UUCUCCGAACGUGUCACGUTT
	NC antisense	ACGUGACACGUUCGGAGAATT
	*si-p62* sense	GGUGCAAAGAUCUCUGUAUTT
	*si-p62* antisense	AUACAGAGAUCUUUGCACCTT
	*si-MyD88* sense	GUACAAGGCAAUGAAGAAATT
	*si-MyD88* antisense	UUUCUUCAUUGCCUUGUACTT
	*si-RIG-I* sense	GCCCAUUUAAACCAAGAAATT
	*si-RIG-I* antisense	UUUCUUGGUUUAAAUGGGCTT
	*si-TRAF3* sense	GGCCGUUUAAGCAGAAAGUTT
	*si-TRAF3* antisense	GGCCGUUUAAGCAGAAAGUTT

### Western blot analysis

Cell samples were lysed using RIPA lysis buffer (89901, Thermo Fisher Scientific) supplemented with phosphatase (B15001; Bimake) and protease inhibitors (SB-WB016). Proteins were transferred to nitrocellulose membranes (10600001, GE Healthcare) following sodium dodecyl sulfate–polyacrylamide gel electrophoresis (SDS-PAGE), followed by membrane blockade using 5% skim milk (36120ES76; YEASEN) and washing with phosphate-buffered saline (PBS) with Tween 20 (PBST). The membrane was probed using primary and secondary antibodies. Proteins were quantified using enhanced chemiluminescence (ECL; Share-bio, SB-WB012).

### RNA isolation and Quantitative real-time polymerase chain reaction (qRT-PCR)

The FastPure Viral DNA/RNA Mini Kit (RC311) or FastPure Cell/Tissue Total RNA Isolation Kit (RC101) (Vazyme Biotech) was applied for total RNA extraction. The RNA was reversed transcribed into cDNA as per the HiScript III RT SuperMix protocol for qPCR (+ gDNA wiper) (R323-01; Vazyme Biotech). The qRT-PCR of the cDNA was conducted as per the SYBR Premix Ex Taq protocol (q711–03; Vazyme Biotech). The list of primer sequences employed in qRTPCR is presented (Table 1).

### Dual-luciferase reporter assay

HEK 293 T cells were seeded into 24-well microplates and transfected with designated plasmids for 24 h. The cells were lysed following specific guidelines (DL101, Vazyme Biotech), enabling Renilla and Firefly luciferase activity assessment. The values were normalized against the internal control (Renilla activity).

### Fluorescence microscopy

After co-transfection with the specified plasmid, HeLa cells were immobilized for 15 min with 4% paraformaldehyde (P6148), followed by permeabilization for 10 min with 0.1% Triton X-100 (T9284) (Sigma-Aldrich). Subsequently, the cells were rinsed three times in PBS and incubated using primary antibody and fluorescent dye-conjugated secondary antibody. The cells were observed using confocal immunofluorescence microscopy (Carl Zeiss).

### Statistical analysis

All experimental data were analyzed using Prism software (GraphPad). Statistical significance was evaluated at the 0.05 level (*P < 0.05, **P < 0.01, and ***P < 0.001, using the two-sided Student *t*-test).

## Results

### SAMSN1 inhibits JEV replication

**Figure 1:**
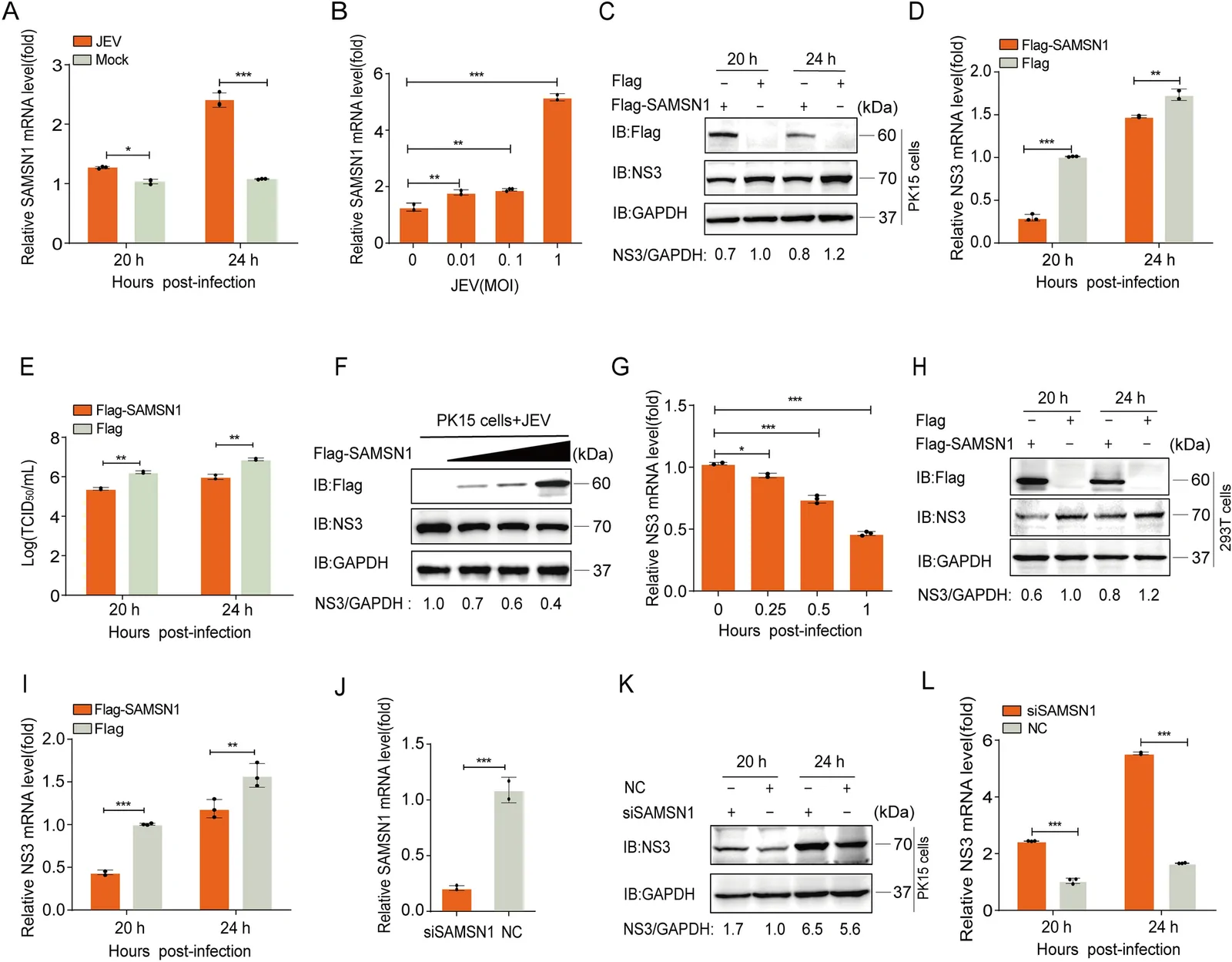
**SAMSN1 inhibits JEV replication.** **A** PK15 cells were infected with JEV (MOI of 1) for 20 and 24 h, and the samples were collected for qRT-PCR assay. **B** Samples were collected for qPCR analysis of SAMSN1 mRNA expression levels after PK15 cells were infected with JEV at different MOIs. **C**–**E** PK15 cells were transfected with Flag-SAMSN1 plasmid and infected with 0.01 MOI of JEV. Samples were gathered and analyzed for viral replication using western blot, qRT-PCR, and TCID_50_. **F**, **G** The best dose of Flag-SAMSN1 was transfected into PK15 cells, then infected with JEV (MOI = 0.01). Western blot and qRT-PCR were carried out to analyze cell lysates. **H**, **I** HEK 293 T cells were transfected with Flag-SAMSN1 and infected with JEV (MOI = 0.01). Viral replication was assessed using western blot and qRT-PCR. **J** PK15 cells were transfected with SAMSN1 siRNA, and interference efficiency was detected via qPCR using porcine SAMSN1 detection primers. **K**, **L** PK15 cells were transfected with SAMSN1 siRNA. Western blot and qRT-PCR were used to analyze cell lysates.

PK15 cells were infected with JEV by setting the multiplicity of infection (MOI) to 1, aiming to understand SAMSN1 regulation. Upregulated messenger RNA (mRNA) expression of SAMSN1 was noted in infected cells relative to uninfected cells (Figure 1A). SAMSN1 mRNA showed a dose-dependent upregulation in infected cells as the MOI increased (Figure 1B). This indicates that JEV infection induces host cell production of SAMSN1 by increasing mRNA levels.

PK15 cells were subjected to SAMSN1 plasmid transfection and JEV infection (MOI = 0.01) to understand SAMSN1 regulation in JEV multiplication. Western blot analysis, qRT-PCR, and 50% tissue culture infectious dose (TCID_50_) assay demonstrated that SAMSN1 overexpression weakened viral replication in PK15 cells (Figure 1C–E; Additional file 1A–B; Additional file 2A–B). When SAMSN1 was ectopically overexpressed, a negative correlation was observed between SAMSN1 expression levels and JEV load (Figure 1F, G). Western blot and qRT-PCR assays revealed that SAMSN1 inhibited JEV replication in HEK 293 T cells (Figure 1H, I). Subsequently, PK15 cells were transfected with SAMSN1 siRNA, which indicated that silencing of SAMSN1 expression enhanced JEV multiplication in these cells (Figure 1J–L), confirming that SAMSN1 hindered JEV replication in PK15 and HEK 293 T cells.

### SAMSN1 interacts with JEV NS1 and NS5 proteins

**Figure 2:**
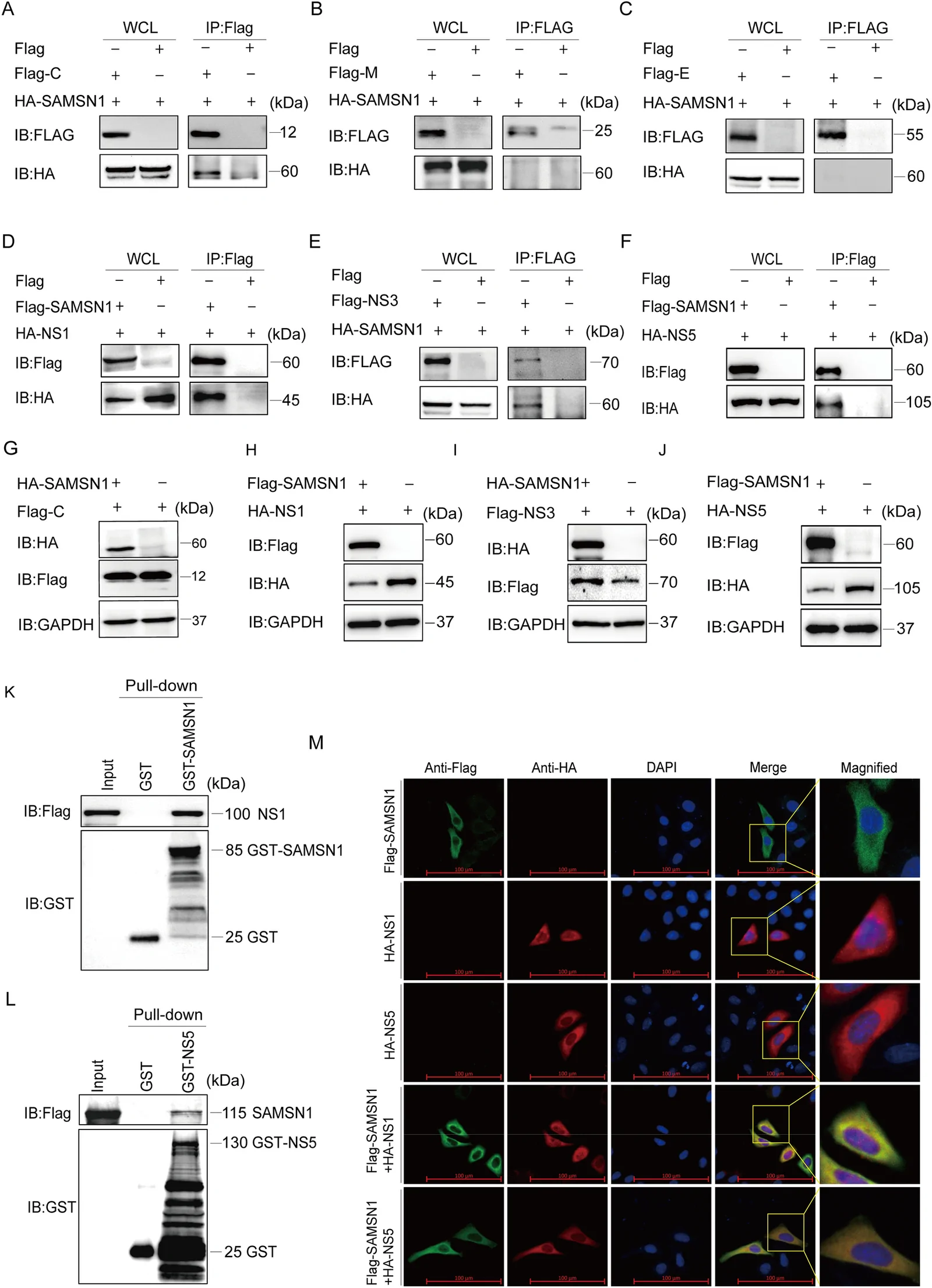
**SAMSN1 interacts with NS1 and NS5.** **A**–**F** HEK 293 T cells were transfected with SAMSN1and JEV-encoded viral protein plasmids; a Co-IP assay was then conducted based on anti-Flag-bound beads, and the samples were analyzed by western blot. **G**–**J** HEK 293 T cells were co-transfected with SAMSN1 and JEV viral protein plasmids. Cell samples were harvested after 24 h, SDS-PAGE was performed, and the results were analyzed. **K**, **L** Prokaryotic pCold-TF-NS1, pCold-TF-SAMSN1, and pCold-GST-NS5 expression plasmids were constructed, and the relationship between SAMSN1, NS1, and NS5 was analyzed by GST pull-down assay. **M** HeLa cells were transfected with Flag-SAMSN1, HA-NS1, and HA-NS5 plasmids, incubated with specific antibodies, and stained with DAPI, then mounted. The cells were observed by confocal immunofluorescence microscope; scale bars:100 μm.

The interactions between SAMSN1 and JEV-encoded viral proteins were examined to identify the molecular mechanism through which SAMSN1 restricts JEV replication. SAMSN1 plasmid and viral protein-expressing plasmids (C, M, E, NS1, NS3, NS5) were transfected into HEK 293 T cells. In accordance with the co-immunoprecipitation (Co-IP) and Peptide MS sequencing (Additional file 6) results, SAMSN1 can precipitate with C, NS1, NS3, and NS5 proteins but does not interact with the M, E protein (Figure 2A–F; Additional file 3A–B). Next, we investigated whether SAMSN1 affects viral protein expression. HEK 293 T cells co-transfected with SAMSN1 and C/NS1/NS3/NS5 plasmids revealed that SAMSN1 reduced the expression of NS1 and NS5, while having no effect on other viral proteins (Figure 2G–J). Therefore, our subsequent experiments focused on the molecular mechanism by which SAMSN1 degrades NS1/NS5. A GST affinity separation assay demonstrated a direct association of SAMSN1 with NS1 or NS5 (Figure 2K, L). The ability of SAMSN1 to co-localize with NS1 or NS5 was examined with confocal microscopy. SAMSN1 co-localized with NS1 or NS5 in the cytoplasm (Figure 2M), indicating that it could directly interact with the NS1 and NS5 JEV proteins.

### SAMSN1 mediates the degradation of JEV NS1 and NS5 protein via the proteasome and autophagy pathway

**Figure 3:**
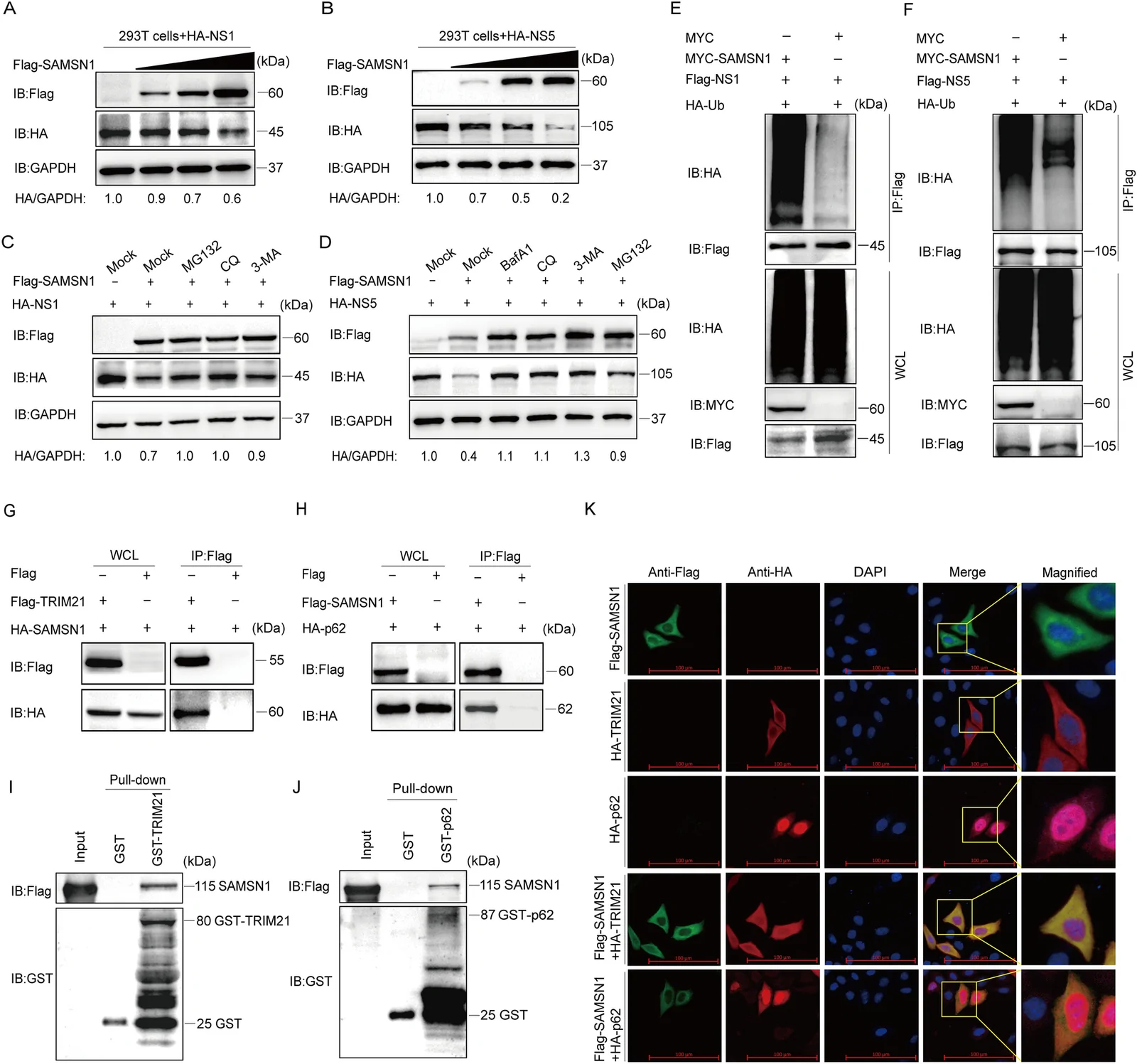
**SAMSN1 mediates degradation of JEV NS1 and NS5 protein via the proteasome and autophagy pathway.** **A**, **B** HEK 293 T cells were co-transfected with varying concentrations of Flag-SAMSN1 and HA-NS1 or HA-NS5 plasmids, and protein samples were collected for western blot analysis. **C**, **D** HEK 293 T cells were transfected with plasmids encoding Flag-SAMSN1 and HA-NS1 or HA-NS5, and treated with bafilomycin A1 (Baf A1), chloroquine (CQ), 3-methyladenine (3-MA), and MG132 9 h prior to cell sample collection. Western blot was used to analyze cell lysates. **E**, **F** HEK 293 T cells were transfected with MYC-SAMSN1, HA-Ub, Flag-NS1, or Flag-NS5. After cell samples were collected, ubiquitination assays were performed using anti-Flag antibodies, and cell lysates were analyzed via western blot. **G**, **H** HEK 293 T cells were co-transfected with SAMSN1, Flag-TRIM21, or HA-p62 plasmids. Co-IP assay was used to explore the interaction between SAMSN1 and TRIM21 or p62. **I**, **J** pCold-TF-SAMSN1, pCold-GST-TRIM21, and pCold-GST-p62 prokaryotic expression plasmids were constructed, and the GST affinity-isolation assay detected GST-TRIM21 or GST-p62 and SAMSN1. **K** Flag-SAMSN1, HA-TRIM21, and HA-p62 plasmids transfected into HeLa cells were subsequently labeled with antibodies for confocal immunofluorescence microscopy. Scale bars:100 μm.

To unravel the mechanism of JEV replication inhibition by SAMSN1, its influence on the NS1 and NS5 protein stability was examined. SAMSN1 and NS1 or NS5 plasmids were co-transfected into HEK 293 T cells. Western blot analysis showed dose-dependent reduction of NS1 and NS5 levels upon SAMSN1 overexpression (Figure 3A, B). The autophagy–lysosome pathway and ubiquitin–proteasome system are the two main degradation mechanisms for intracellular proteins [23]. To examine the NS1 and NS5 degradation mechanism by SAMSN1, protease suppressor MG132 and autophagy suppressors chloroquine (CQ), 3-methyladenine (3-MA), and bafilomycin A1(Baf A1) were applied. The results suggested that autophagy and protease inhibitors inhibited SAMSN1-induced NS1 and NS5 degradation (Figure 3C, D). Ubiquitin–proteasome and autophagy–lysosome degradation requires ubiquitination of substrate proteins. SAMSN1 overexpression prominently increases NS1 and NS5 protein ubiquitination (Figure 3E, F). Selective autophagy is a catabolic degradation process, wherein specific cellular components are targeted to autophagosomes. E3 ubiquitin ligases ubiquitinate specific substrates, and they are recognized by the cargo receptors. To identify the ubiquitinating enzyme and cargo receptor engaged in the SAMSN1-mediated NS1 and NS5 protein degradation, HEK 293 T cells were co-transfected with plasmids encoding ubiquitinating enzyme, cargo receptor, and SAMSN1. Co-IP assays revealed interactions between SAMSN1 and E3 ubiquitin ligase TRIM21 and cargo receptor p62 (Figure 3G, H). GST affinity separation experiments confirmed a direct interaction between SAMSN1 with TRIM21 or p62 (Figure 3I, J). Immunofluorescence confocal microscopy results confirmed the co-localization of SAMSN1 with TRIM21 and p62 (Figure 3K). In addition, the E3 ubiquitin ligase TRIM21 suppresses JEV replication (Additional file 4A–C); when TRIM21 expression is disrupted, JEV replication is enhanced (Additional file 4D–F). TRIM21 expression is upregulated during JEV infection (Additional file 4G–I). The results suggest that SAMSN1 stimulated NS1 and NS5 degradation through the proteasome and autophagy pathway.

### JEV NS1 and NS5 proteins are degraded via the SAMSN1-TRIM21-p62 autophagosome pathway

**Figure 4:**
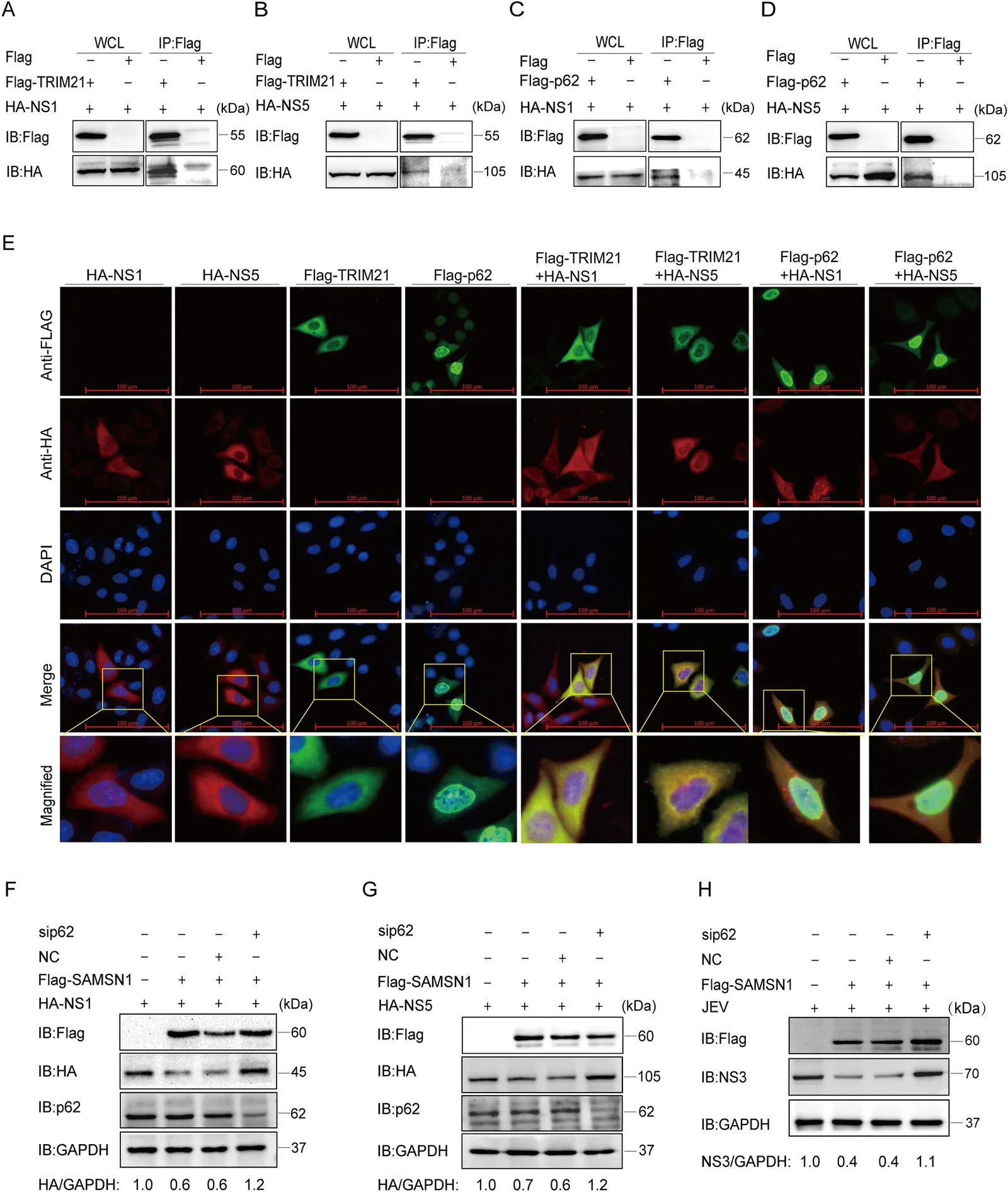
**JEV NS1 and NS5 proteins were degraded by the SAMSN1-TRIM21-p62 autophagosome pathway.** **A**, **B** After co-transfection with Flag-TRIM21 and HA-NS1 or HA-NS5 plasmids, the HEK 293 T cells were collected for Co-IP experiments. **C**, **D** After the HEK 293 T cells were co-transfected with Flag-p62-, HA-NS1-, or HA-NS5-encoding plasmids, anti-Flag bound beads were used for Co-IP assays. **E** HeLa cells were transfected with HA-NS1, HA-NS5, Flag-TRIM21, and Flag-p62 plasmids; after cell sample collection, cells underwent fixation, permeabilization, and blocking. Specific antibodies were then applied for incubation, followed by DAPI staining and mounting. Cells were visualized using confocal immunofluorescence microscopy. **F**, **G** HEK 293 T cells were transfected with Flag-SAMSN1, p62 siRNA, HA-NS1, or HA-NS5 plasmids, and cell samples were collected and analyzed by western blot using a specific p62 antibody. **H** When PK15 cells reached the appropriate density, p62 siRNA was transfected into the cells; 24 h later, the cells were infected with JEV. Samples were collected at appropriate time points, and the results were analyzed by western blot.

NS1, a glycosylated protein with various oligomeric forms in the viral replication cycle, is essential for flavivirus genome replication [24]. Its RNA-reliant RdRp structural domain makes NS5 an antiviral target [25]. As SAMSN1 mediates NS1 and NS5 protein degradation via TRIM21 and p62 interaction, NS1 and NS5 can interact with TRIM21 and p62. Co-transfection of Flag-TRIM21, HA-NS1, or HA-NS5 plasmids into HEK 293 T cells demonstrated the binding between the proteins in co-IP experiments (Figure 4A, B), especially between p62 and NS1 or NS5 protein (Figure 4C, D). Confocal immunofluorescence observations revealed TRIM21 and p62 co-localized with NS1 or NS5 in the cytoplasm (Figure 4E). To further investigate the crucial role of autophagy protein degradation in SAMSN1-induced degradation of JEV NS1 and NS5 proteins, we transfected p62 siRNA to inhibit the autophagy degradation pathway. Reduced p62 protein expression suppressed the NS1 and NS5 degradation by SAMSN1 (Figure 4F, G; Additional file 5A). We hypothesize that SAMSN1 mediates NS1 protein degradation through the SAMSN1-TRIM21-p62 protein degradation pathway, thereby affecting viral replication. To validate this conclusion, p62 expression of PK15 cells was inhibited, and cells were infected with JEV. The results showed that viral replication was enhanced with interference of p62 expression (Figure 4H). The results indicate that autophagy protein degradation is implicated in the NS1 and NS5 degradation by SAMSN1.

### SAMSN1 promotes IFN expression through interaction with RIG-I and MyD88

**Figure 5:**
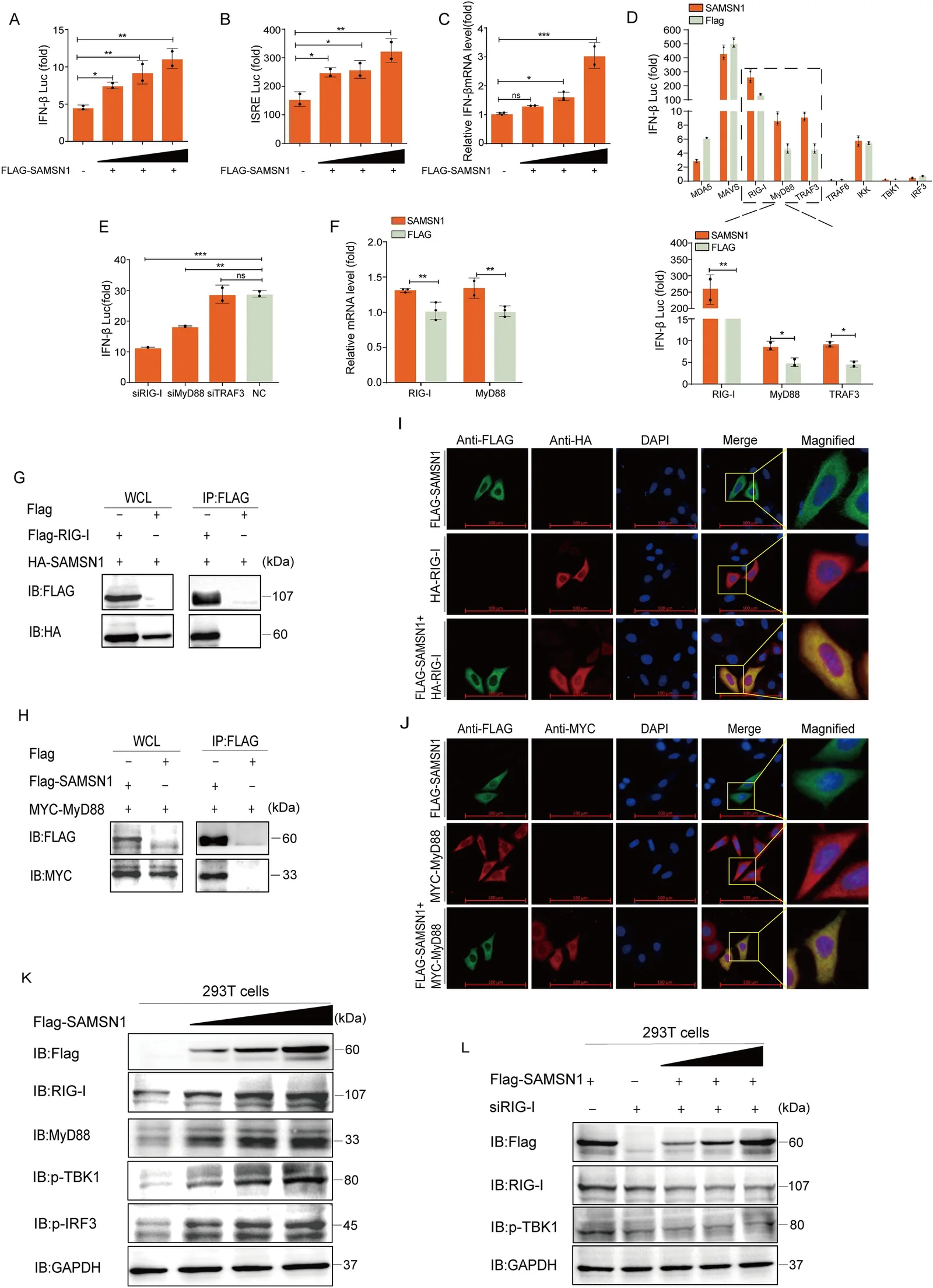
**SAMSN1 promotes IFN expression by interaction with RIG-I and MyD88.** **A**, **B** HEK 293 T cells were transfected with different doses of Flag-SAMSN1 and the empty vector, cell samples were collected 24 h later, and IFN-β and ISRE dual luciferase activity was measured. **C** HEK 293 T cells were transfected with different doses of Flag-SAMSN1 and empty vector, cell samples were gathered at 24 h post-transfection, and IFN-β mRNA levels were analyzed by qRT-PCR. **D** HEK 293 T cells were co-transfected with SAMSN1, IFN-β luciferase reporter, and plasmids encoding various signaling molecules to examine the IFN-β dual luciferase activity. **E** Flag-SAMSN1, RIG-I siRNA, MyD88 siRNA, and TRAF3 siRNA were co-transfected into HEK 293 T cells to examine the IFN-β dual luciferase activity. **F** HEK 293 T cells were transfected with plasmids encoding Flag-SAMSN1 and empty vector, and RIG-I and MyD88 mRNA levels were analyzed by qRT-PCR. **G**, **H** Plasmids encoding HA-SAMSN1, Flag-RIG-I, and MYC-MyD88 were transfected into HEK 293 T cells in a Co-IP assay. **I**, **J** An immunofluorescence assay was performed to explore the co-localization of Flag-SAMSN1, HA-RIG-I, or MYC-MyD88 through laser confocal experiments. **K** The Flag-SAMSN1 expression plasmid and empty vector at increasing doses was transfected into HEK 293 T cells, and western blot analysis was performed to detect the protein expression levels of RIG-I, MyD88, p-TBK1, and p-IRF3. **L** Flag-SAMSN1 expression vector (wedge) at increasing dose was co-transfected with RIG-I siRNA into HEK 293 T cells. Western blot was performed to analyze the cell lysates.

During a viral infection, the host’s innate immunity is the first line of defense against viral invasion and multiplication [26]. In order to determine whether SAMSN1 inhibits JEV by affecting the IFN-mediated antiviral reactions, IFN-β promoter and IFN stimulated response element (ISRE)-driven luciferase reporter assay was conducted. SAMSN1 induced IFN-β activation in a dose-dependent manner (Figure 5A, B). with increased SAMSN1 expression, the IFN mRNA level increased proportionally, as seen in luciferase reporter assay findings (Figure 5C). To clarify how SAMSN1 promotes IFN expression, HEK 293 T cells were co-transfected with SAMSN1 plasmid and a plasmid encoding key innate antiviral response-related signaling proteins. SAMSN1 increased the RIG-I-, MyD88-, and TRAF3-elicited luciferase reporter activity (Figure 5D). Interference or overexpression of RIG-I, MyD88, and TRAF3 in HEK 293 T cells indicates that RIG-I and MyD88 participate in regulating IFN expression by SAMSN1 (Figure 5E, F). Co-IP and confocal immunofluorescence experiments revealed interaction of SAMSN1 with RIG-I and MyD88, and SAMSN1 localization (Figure 5G–J). SAMSN1 upregulated the endogenous RIG-I and MyD88 in HEK 293 T cells efficiently and in a dose-dependent manner (Figure 5K). SAMSN1 lost the upregulating effect of IFN in cells co-transfected with RIG-I siRNA (Figure 5L; Additional file 5B–D). These results indicate that SAMSN1 interacts with RIG-I and MyD88, activating the IFN signaling pathway, thus preventing JEV infection.

## Discussion

**Figure 6:**
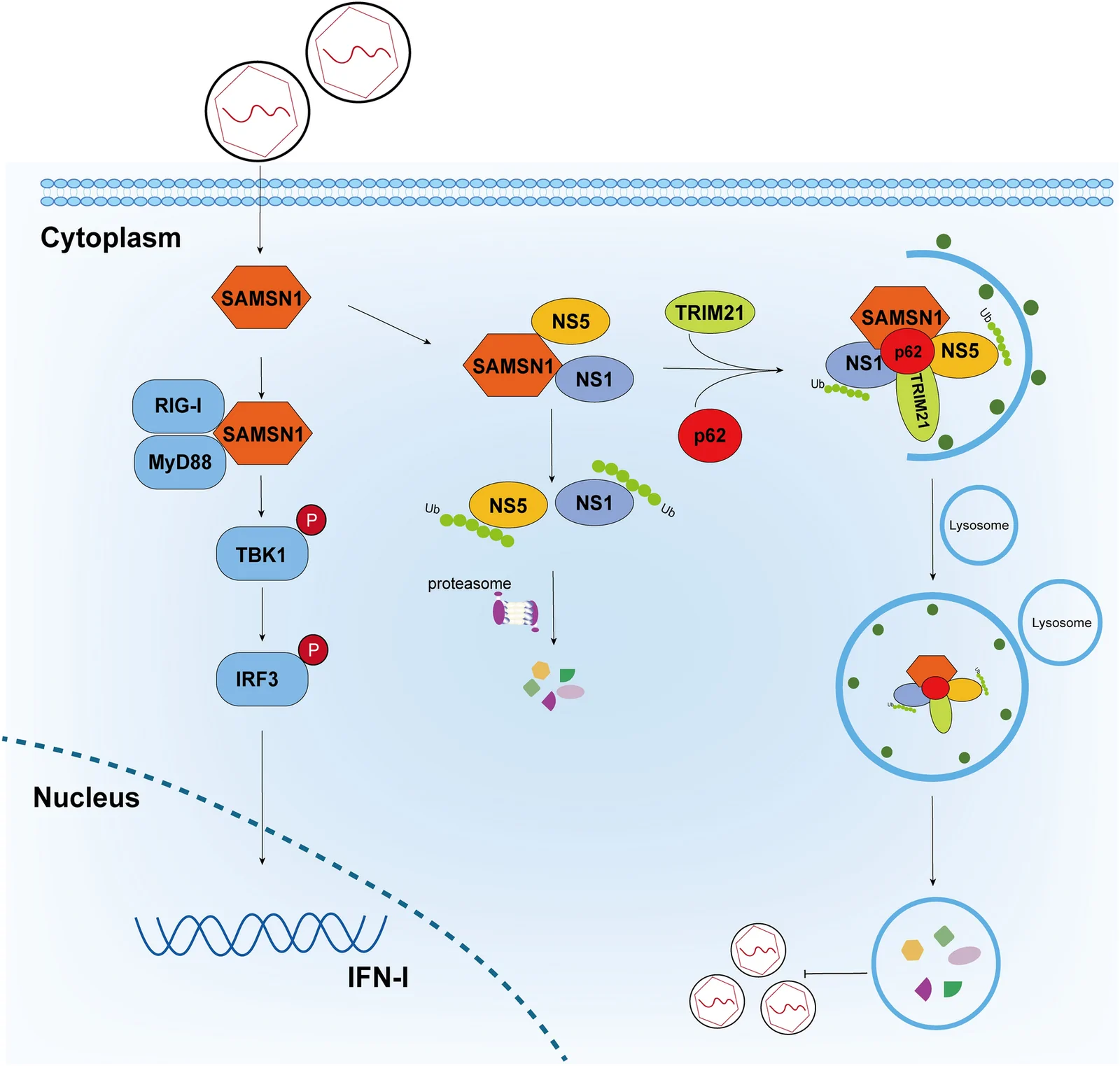
**Mechanism of SAMSN1-TRIM21-NS1/NS5-p62 inhibition of JEV proliferation.** Upon JEV infection, SAMSN1 recruits TRIM21 (E3 ubiquitin ligase) to the JEV NS1 and NS5 proteins for mediating ubiquitination of nonstructural proteins, subsequently recognized by p62 (cargo receptor) and delivered to the lysosome for degradation. Additionally, IFN expression stimulated by SAMSN1 interacts with RIG-I and MyD88 to facilitate the host anti-JEV defense responses.

Japanese encephalitis (JE) causes inflammation of the central nervous system. It can affect humans and certain animals. At present, there are no specific antiviral drugs available against the virus. Flaviviruses like JEV pose a serious global health concern. This study identified SAMSN1, a novel antiviral host factor. SAMSN1 can restrict JEV infection via the proteasome or autophagy degradation pathway. It recruits E3 ligase TRIM21 and cargo receptor p62 to mediate JEV NS1 and NS5 protein degradation. Additionally, SAMSN1 positively regulates the IFN response by binding RIG-I and MyD88, demonstrating a novel immunomodulatory mechanism of SAMSN1 (Figure 6).

The life cycle of the flavivirus is highly dependent on the key effects exerted by the host’s defense mechanisms. Identifying factors involved in viral replication and virus–host interaction is important in developing innovative antiviral therapies [27]. SAMSN1, an adaptor and scaffold protein, encompasses SH3 and sterile alpha motif (SAM) domains that prevail in hematopoietic cells, and mediate differentiation and activation of β-cells (9). Upregulation of SAMSN1 seems to be a risk factor for glioblastoma multiforme patients [28]. SAMSN1 is engaged in regulating actin cytoskeleton remodeling, involving cell adhesion and migration [29, 30]. Our current study found that SAMSN1 possesses antiviral activity, with its mRNA levels significantly upregulated following viral infection. We hypothesize that during JEV infection, the host recognizes the virus and activates innate immune responses, thereby upregulating SAMSN1 expression and enhancing its antiviral function. Further investigations revealed that SAMSN1 exerts antiviral effects by mediating JEV nonstructural protein degradation via proteasome and autophagy pathways. SAMSN1 inhibits viral infections through IFN upregulation. These findings highlight the previously unknown roles of SAMSN1.

Flavivirus nonstructural proteins are associated with its replication. This process is dependent on the membrane-bound multiprotein complex assembly [31, 32]. The nonstructural proteins play a role in remodeling the internal cellular organization of mature polyproteins, RNA replication, and escaping host immune response [25]. NS1 is a highly conserved dimeric protein, which exerts crucial effects on secretion, virulence, and replication [33]. NS5, the largest nonstructural glycoprotein, is an RNA-reliant RdRp and an RNA MTase. It protects the viral genome through RNA capping and facilitates multiprotein translation. NS5 interacts with various proteins in the human host, like proteins in the JAK-STAT axis, to disrupt antiviral interferon signaling [34]. In the present study, we found that SAMSN1 interacted with NS1 and NS5 to inhibit JEV proliferation.

The autophagy–lysosomal pathway acts as a mechanism mediating the degradation of ubiquitinated protein aggregates when molecular chaperone-assisted proteasomal degradation of misfolded proteins fails. This results in protein aggregation, leading to proteasome inactivation and inducing cytotoxicity [35]. TRIM44 (tripartite motif-containing 44) refers to a novel link between the autophagy degradation pathway and the ubiquitin-proteasome system (UPS). UPS pathway inhibition causes TRIM44 upregulation, stimulating protein aggregate clearance by binding to the K48 ubiquitin chain on the protein [36]. TRIM proteins completely utilize their E3 ubiquitin ligase activity. TRIM52 plays an anti-JEV role through flavivirus NS2A targeting and degradation [37]. TRIM21 facilitates rabies virus proliferation by IRF7-ubiquitinated degradation of [38]. TRIM69 suppresses DENV multiplication by nonstructural protein ubiquitination [39]. Studies have shown that during JEV infection, multiple interferon-stimulated genes (ISGs) are activated, including TRIM21 [40]. In this study, SAMSN1 mediates the degradation of JEV nonstructural proteins by recruiting the E3 ubiquitin ligase TRIM21. p62 is a cytoplasmic regulator molecule, which connects ubiquitinated proteins to autophagy [41]. p62 is associated with ubiquitin-positive inclusion body formation and enhances autophagic degradation by binding to LC3II. According to our findings, SAMSN1 recruits TRIM21, and facilitates lysosomal transport of JEV proteins for degradation through p62. p62 interacts with nonstructural proteins forming protein complexes involved in autophagic degradation.

The innate immune response eliminates viruses and controls viral proliferation during the early infection phase. Toll-like receptor (TLR)-, nod-like receptor (NLR)-, rig-like receptor (RLR)-, or DNA receptor CGAS-mediated innate immunity is the host’s first line of defense against a viral invasion. These receptors recruit downstream molecules (e.g., TBK1, MAVS, or RIP2) upon viral invasion, triggering IRF3 activation [18]. A variety of animal and cell line models have acknowledged the importance of innumerable pattern recognition receptors (PRRs) in identifying JEV components to initiate innate immunity [42]. TLR-3, RIG-I, and MDA-5 activation in JEV-infected microglial and neuronal cells is critical for viral suppression, since their deletion leads to weakened immunity and higher viral load [43, 44]. RIG-I is a prime PRR responsible for viral transcriptional intermediate and single-stranded RNA recognition. RIG-I interacts with mitochondrial antiviral signaling (MAVS), and IRF3 and IRF7, two interferon regulatory factors, are phosphorylated [45]. In the present study, SAMSN1 upregulated RIG-I and MyD88 levels, which in turn regulated TBK1, phosphorylated IRF3, and induced IFN-I secretion. This function further contributes to the anti-JEV activity of SAMSN1.

To summarize, SAMSN1 inhibits JEV infection through the initiation of the SAMSN1-TRIM21-NS1/NS5-P62 pathway during infection. Additionally, SAMSN1 positively regulated IFN responses by binding RIG-I and MyD88, providing a theoretical basis for combating flaviviruses.

## Supplementary Information

**Additional file 1. Evaluating the cytotoxicity induced by overexpression plasmids and siRNAs.(A-B)** HEK 293T cells transfected with the Flag-SAMSN1 plasmid or SAMSN1 siRNA. After 24 h, use the CCK-8 kit were used to measure the cell viability.

**Additional file 2. SAMSN1 inhibits JEV replication.(A-B)** PK15 cells were transfected with HA-SAMSN1 and PXJ41 empty vector plasmids. After 24 h, cells were infected with JEV. Cell samples and viral supernatants were collected at 36 and 48 hpi, an analyzed via western blot and qPCR.

**Additional file 3. SAMSN1 interacts with NS1 and NS5. (A-B)** HEK 293T cells were transfected with Flag-SAMSN1plasmids and HA-NS1/HA-NS5, and the samples were harvested at 24 hours post-transfection anti-HA Rabbit antibody was incubated with protein beads at 4 °C for 1-2 h, followed by incubation with protein samples at room temperature for 1-2 h. For western blot analysis, a mouse anti-HA antibody was used to detect protein bands.

**Additional file 4. TRIM21 Inhibits JEV Proliferation.(A-C)** PK15 and HEK 293T cells were overexpressed Flag-TRIM21, 24h later, cells were infected with JEV (0.01 MOI). Protein samples and viral supernatants were collected at 20 and 24 h post-infection. Detection was performed via western blot and qPCR, with results analyzed. **(D-F)** Synthesized porcine-origin TRIM21 siRNA at Shanghai Gema Pharmaceutical Technology Co., Ltd. Following protocol dilution and mixing, PK15 cells was transfected siRNA, which were infected with JEV (0.01 MOI). Samples were collected at appropriate time points. TRIM21 infection efficiency was assessed via western blot, while NS3 mRNA levels were measured by qPCR to evaluate viral replication capacity. **(G-I)** PK15 cells were infected with JEV. Protein samples were collected at 12 and 24 h post-infection to detect TRIM21 protein and mRNA levels. Concurrently, PK15 cells were infected with JEV at different MOIs, and protein samples were collected to measure NS3 mRNA levels.

**Additional file 5. Assessment of the interference efficiency of sip62 and siRIG-I, as well as the cytotoxicity ofsiRNA.(A)** HEK 293T cells were transfected with p62 siRNA, and detect interference efficiency via qPCR. **(B)** HEK 293T cells transfected with RIG-I siRNA. After 24 h, use the CCK-8 kit were used to measure the cell viability. **(C)** HEK 293T cells were transfected with RIG-I siRNA, and detect interference efficiency via qPCR. **(D)** HEK 293T cells were transfected with RIG-IsiRNA interfering RNA, followed by overexpression of Flag-SAMSN1 plasmid at different concentrations. After 24 h, cell samples were collected and analyzed via western blot to detect the levels of RIG-I, p-TBK1, and p IRF3 proteins.

**Additional file 6. Peptide MS sequencing.** HEK293T cells were transfected with the Flag-NS3 plasmid. Cells were harvested at 24 h and IP experiment was performed. Then the protein mixture was sent to Shanghai houji biological company (China) for peptide mass spectrometry (MS) sequencing analysis.

## Data Availability

All the data generated during the current study are included in the manuscript.
